# Improved Chromatographic Separation of Sitagliptin Phosphate and Metformin Hydrochloride

**Published:** 2015-12

**Authors:** Moataz S. Hendy

**Affiliations:** Department of Chemistry, Faculty of Pharmacy, British University in Egypt, El-Sherouk city, Cairo, Egypt

**Keywords:** Sitagliptin, Metformin, UPLC, Column temperature, factorial design

## Abstract

New UPLC method was developed for determination of sitagliptin and metformin using Symmetry C_18_ column (100 mm × 2.1 mm, 2.2 μm) and isocratic elution (methanol 20%), pH (3.5) as a mobile phase. The ultraviolet detector was operated at 220 nm and the column temperature was 50°C. Linearity parameters were acceptable over the concentration ranges of 2-12 μgml^-1^ and 5-35 μgml^-1^ for sitagliptin and metformin, respectively. The variables were premeditated to adjust the chromatographic conditions using design of experiment. The proposed method was proved to be accurate for the quality control of the mentioned drugs in their pharmaceutical dosage form.

## INTRODUCTION

**Figure 1:**
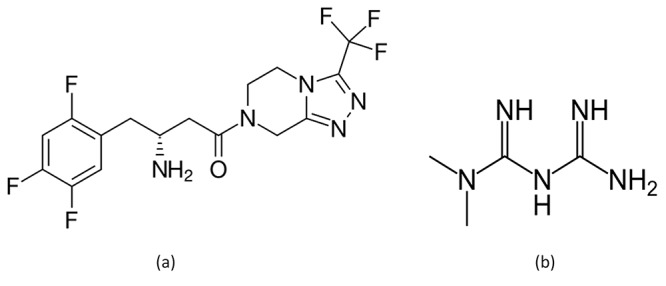
Chemical structures of sitagliptin (a) and Metformin (b).

Sitagliptin (SGN), 1,2,4- triazolo [4,3-a] pyrazine,7- [(3*R*)-3-amino-1-oxo-4- (2,4,5-trifluorophenyl) butyl] -5,6,7,8- tetrahydro-3- (trifluoromethyl) phosphate (Figure 1a) and metformin hydrochloride (MET), *N*,*N*-dimethylimidodicarbonimidic diamide (Figure 1b) are oral hypoglycemic drugs. Some well established liquid chromatographic methods with ultraviolet detection were described for simultaneous determination of SGN and MET in tablets (1-5) and also some spectrophotometric methods were reported (6-8). The aim of the work is to develop a new UPLC method with many advantages over the routine HPLC methods found in the literature (1-5); using simple mobile phase as methanol 20% without ion pairing reagent or buffer, decreased cost as UPLC is more economic than HPLC consuming less organic solvent and less time.

## EXPERIMENTAL

### Instrumentation

The liquid chromatography consisted of a Thermo Fisher UPLC Model Ultimate 3000 (USA), a Symmetry^®^ C_18_ column (100 mm × 2.1 mm, 2.2 μm) equipped with a Diode Array detector (DAD-3000RS, USA) and an auto sampler (WPS-3000TRS, USA).

### Reagents, reference samples and working solutions

Pharmaceutical grade sitagliptin phosphate monohydrate and metformin hydrochloride certified to contain 99.7 % and 99.8 % respectively, Janumet^®^ tablets nominally containing 64.25 mg of sitagliptin phosphate monohydrate and 500 mg of metformin hydrochloride per tablet were kindly supplied from Merck Sharp and Dohme Co. (Egypt). HPLC grade methanol was purchased from Fisher Scientific (UK). Working solutions of SGN (20 μg mL^-1^) and MET (50 μg mL^-1^) were prepared separately in the mobile phase.

### Sample preparation

Ten tablets of Janumet^®^ were weighed and powdered. An accuratelyweighed amount equivalent to 4 mg of SGN and 31 mg of MET were made up to100 mL with methanol and sonicated to dissolve. The solutions were filtered followed by serial dilutions using the mobile phase to be (4 μg/ml SGN) and (31 μg/ml MET).

### Adjustment of chromatographic conditions using factorial design

**Figure 2:**
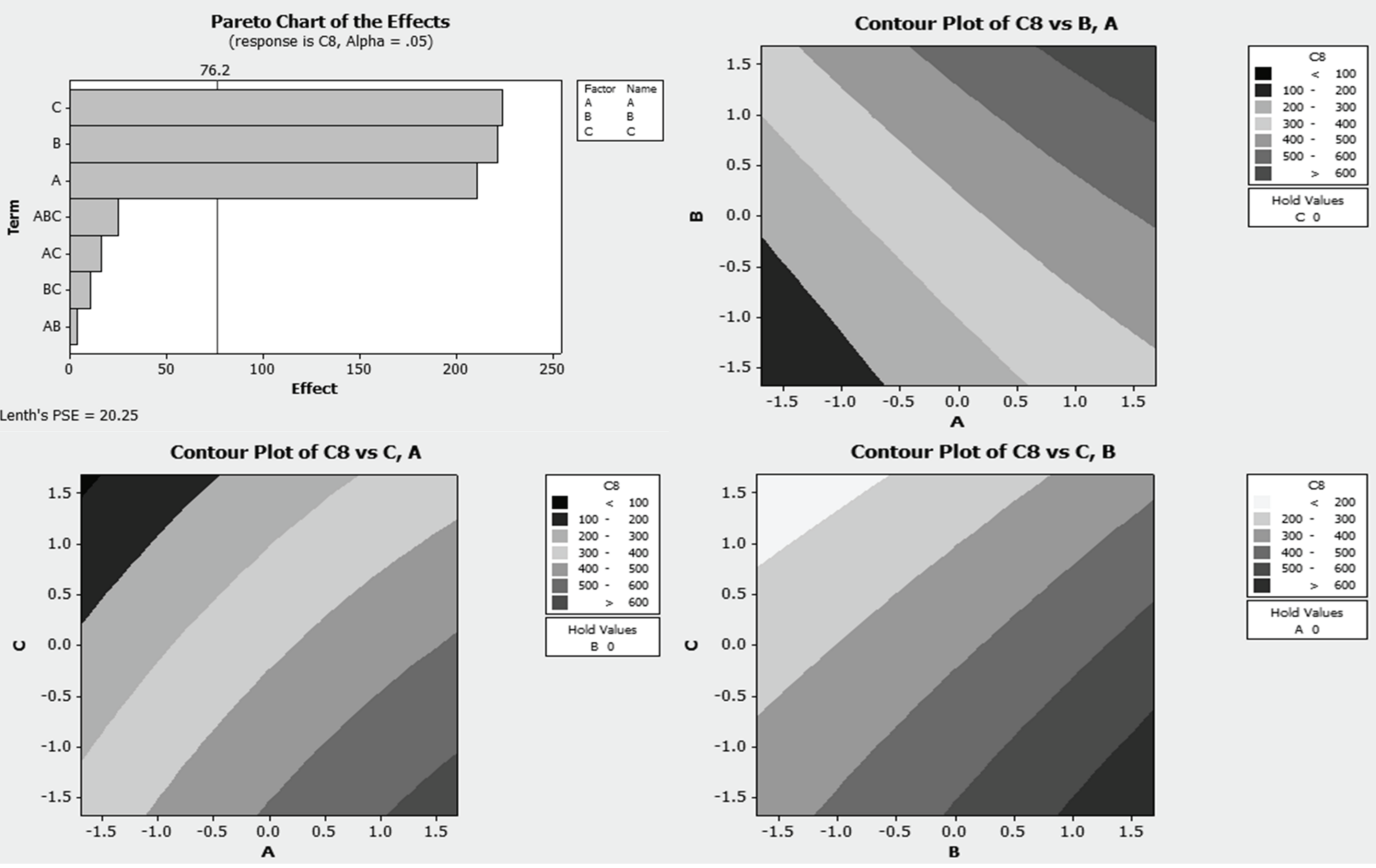
Pareto chart and contour plots of the investigation.

Optimization of the chromatographic conditions was performed by design of experiment using Minitab® program. In the first step of the factorial design, experimental design was used to detect variables which have imperative influence on the chromatographic performance (Table 1). Two levels were used. A graphical display of each factor was given in a Pareto chart. A factor was considered as “statistically significant” if its effect exceeded a line in the Pareto chart (Figure 2). In the second step of the factorial design, response surface methodology was used where the effect of two variables can be represented as a surface in three-dimensional space and the influence of two variables on the response can be clearly seen in the investigated region (Figure 2).

**Table 1:** Investigated variables

Variables (6 factors)	Investigated levels (2 levels with -1,1 values)	
	Low level (-1)	High level (+1)

A: pH of the mobile phase	2.5	3.5
B: Column temperature	20	50
C: Methanol ratio in mobile phase	20%	60%

**Figure 3:**
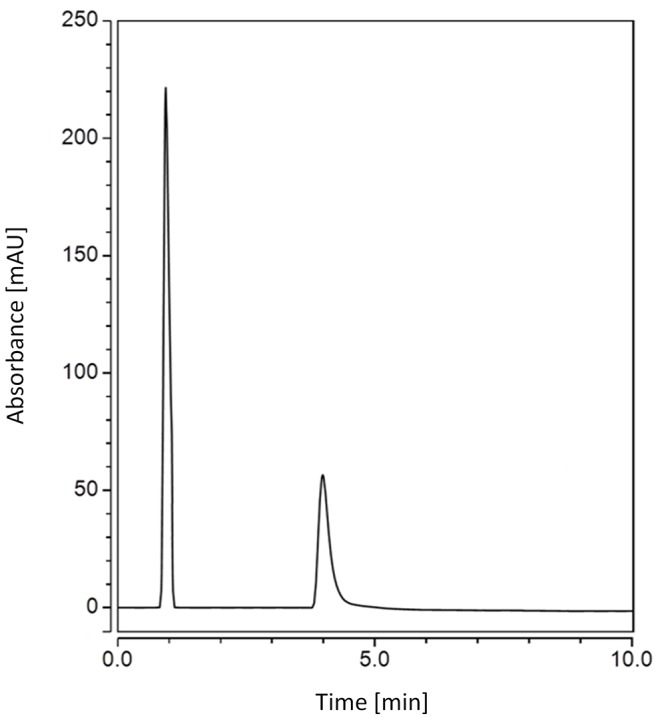
UPLC chromatogram of Janumet^®^ tablet containing sitagliptin phosphate monohydrate (4 μg mL^-1^) at 1.1 min and metformin hydrochloride (31 μg mL^-1^) at 4.2 min.

According to Figure 2, pH was found optimum at 3.5, Column temperature was found optimum at its higher level (50°C) and finally methanol ratio in the mobile phase was found to be at its minimum level. Several columns had been used in the literature for determination of gliptins and metformin. Cyano column was used with good results for some gliptins (9-10) but C_18_ column also was used successfully for their determination (11). According to the literature, C_18_ column was used usually for the simultaneous determination of MET in mixtures with gliptins (12), glitazones (13) and gliflozins (14) so C_18_ column was selected for the enhancement described in this method. pH of the mobile phase (methanol 20%) was adjusted to 3.5 using acetic acid. UV detection at 220 nm was selected. Adjusting column temperature to 50°C enhanced the resolution of peaks. The flow rate was selected to be 0.4 mL min^-1^ and the injection volume was 10 micro liters. Good resolution between peaks was obtained (Figure 3).

### Procedure

**Linearity.** Aliquots of working solutions equivalent to 20-120 μg and 50-350 μg for SGN and MET, respectively were transferred separately into a series of 10 mL volumetric flasks, completed to volume with the mobile phase and only ten micro liters was injected. A calibration curve was obtained by plotting area under the peak of the corresponding drug against its concentration.

**Assay of SGN and MET in lab prepared mixtures (accuracy) and Janumet^®^ tablets.** Different ratios of SGN and MET were prepared (SGN: MET, 5:10 μg/mL, 5:5 μg/mL and 10:5 μg/mL). For the determination of SGN and MET in Janumet^®^ tablets, the sample solution was prepared as under 2.3. Only ten micro liters was injected and the concentrations of SGN and MET in lab prepared mixtures and tablets were calculated using their calibration equations.

## RESULTS AND DISCUSSION OF THE VALIDATION PARAMETERS

### System suitability tests

System suitability tests including number of theoretical plates, tailing factor and resolution between peaks were calculated for the proposed method. The results of these tests are listed in Table 2.

**Table 2:** System suitability tests

Item	MET	SGN

N (Number of theoretical plates)	1241	1847
T (Tailing factor)	1.00	1.18
R (Resolution between two consecutive peaks)	8.1	
RSD% of 6 injections		
Peak area	0.25	0.23
Retention time	0.35	0.16

N, Number of theoretical plates; T, Tailing factor; R, Resolution between two consecutive peaks; RSD, Relative standard deviation.

### Linearity

A linear relationship between area under the peak and the concentration of each drug was obtained, regression parameters were computed and the linearity of the calibration curves were validated by low values of limit of detection (LOD) and limit of quantification (LOQ) as listed in (Table 3).

### Accuracy

Accuracy of the results was confirmed by the recovery percent of each drug in laboratory prepared mixture. The results including the mean of the recovery and standard deviation are shown in (Table 3).

**Table 3:** Results obtained by the proposed UPLC method

Item	SGN	MET

UPLC-UV detection	220 nm	220 nm
Retention time (min)	4.2	1.1
Linearity	2-12 μg.ml^-1^	5-35 μg.ml^-1^
Regression equation	AUP = 5.1012 C_μg/ml_ + 0.2431	AUP = 1.4275 C_μg/ml_ + 0.3974
(r^2^)	0.9997	0.9995
Accuracy (mean ± SD)	100.23 ± 0.56	99.84 ± 1.34
Dosage form (mean ± SD)	101.22 ± 0.33	98.88 ± 0.74
LOD μg.ml^-1^	0.12	1.64
LOQ μg.ml^-1^	0.36	4.92
Intraday %RSD	0.24-0.39	0.27-0.42
Interday %RSD	0.20-0.42	0.31-0.48
Standard error of the estimation	0.19	0.71

### Precision

The three lab prepared mixtures (SGN: MET, 5:10 μg/mL, 5:5 μg/mL and 10:5 μg/mL) were analyzed three times, within the same day and also on three successive days. The %RSD was calculated and found to be less than 2 % in the three concentrations, as shown in (Table 3).

### Robustness

The flow rate of the mobile phase was changed from 0.40 mL min^-1^ to 0.41 mL min^-1^ and 0.39 mL min^-1^. The organic strength was changed by % ± 1. And the pH value of the buffer was varied from 3.5 to 3.4 and 3.6. There is no significant difference in the results indicating good robustness of the method.

## CONCLUSION

The proposed method proved to be simple and reproducible for determination of SGN and MET. The method was validated showing good results for all the parameters tested. The developed method can be conveniently used by quality control laboratories.
